# Revisiting the link between body and agency: visual movement congruency enhances intentional binding but is not body-specific

**DOI:** 10.1038/s41598-017-18492-7

**Published:** 2018-01-09

**Authors:** Regine Zopf, Vince Polito, James Moore

**Affiliations:** 1 ARC Centre of Excellence in Cognition and its Disorders, Macquarie University, Sydney, Australia; 2 Department of Cognitive Science, Macquarie University, Sydney, Australia; 3 Perception in Action Research Centre, Faculty of Human Sciences, Macquarie University, Sydney, Australia; 4 Department of Psychology, Goldsmiths, University of London, London, UK

## Abstract

Embodiment and agency are key aspects of how we perceive ourselves that have typically been associated with independent mechanisms. Recent work, however, has suggested that these mechanisms are related. The sense of agency arises from recognising a causal influence on the external world. This influence is typically realised through bodily movements and thus the perception of the bodily self could also be crucial for agency. We investigated whether a key index of agency – intentional binding – was modulated by body-specific information. Participants judged the interval between pressing a button and a subsequent tone. We used virtual reality to manipulate two aspects of movement feedback. First, *form*: participants viewed a virtual hand or sphere. Second, *movement congruency*: the viewed object moved congruently or incongruently with the participant’s hidden hand. Both factors, *form* and *movement congruency*, significantly influenced embodiment. However, only *movement congruency* influenced intentional binding. Binding was increased for congruent compared to incongruent movement feedback irrespective of form. This shows that the comparison between viewed and performed movements provides an important cue for agency, whereas body-specific visual form does not. We suggest that embodiment and agency mechanisms both depend on comparisons across sensorimotor signals but that they are influenced by distinct factors.

## Introduction

Typically, we perceive ourselves as distinct from others and the environment, and this distinction is crucial for our interactions with the external world. Two key aspects of self-perception are the sense of embodiment and the sense of agency^1,2^. The sense of embodiment involves the ability to perceive that one’s own body belongs to oneself and to know its location^3^. Whereas, the sense of agency involves the ability to perceive that one is initiating, executing and controlling one’s own voluntary actions and their effects^4,5^.

Embodiment and agency have typically been thought to rely on mostly independent mechanisms^6,7^. However, recent findings have also suggested some links between these two self-perception components^5,6,8–14^. Understanding similarities and differences between embodiment and agency mechanisms will have important implications for understanding how both can be altered in clinical disorders such as schizophrenia^4,15–19^, or when interacting with technological tools, such as virtual reality and prosthesis^5,8,20–23^.

The cognitive mechanisms underlying embodiment and agency might be related. Our sense of agency arises from recognising our causal influence on the external world. Typically, this influence is realised through bodily movements that link our intentions to subsequent effects or outcomes^5^. Thus, the perception of the bodily self could be crucial for both embodiment and agency mechanisms. Consequently, one approach to further understand the links between embodiment and agency is to investigate the extent to which both are affected by specific bodily self-cues^5,6,9,24^.

Bodily self-cues have been studied extensively within the context of the Rubber Hand Illusion (RHI) paradigm and its variants. In this paradigm an artificial hand is viewed next to one’s own hand, which is hidden from view. The artificial hand may be a model hand, robotic, or a 2D- or 3D-image on a computer or head-mounted screen^6,8,12,25,26^. When the artificial hand is touched or made to move at the same time as the participant’s hand, this leads to the sense of embodiment. Participants may report feeling body ownership for the artificial body and feeling that their real limb is located closer in space to the artificial limb^6,12,25,27^. Crucially, experimental paradigms, such as the RHI, allow researchers to manipulate bodily self-cues by creating conflicts between visual and non-visual signals.

RHI research has shown that one bodily self-cue that is important for embodiment is the temporal congruency between multisensory signals^27^. For example, when one views an artificial or virtual hand that moves at the same time as one’s own hand, then this can induce strong feelings of embodiment for the external hand^6,8^. However, when a delay between performed movement and visual movement feedback is presented, embodiment is reduced^6,8^. In contrast, agency is typically less affected by temporal delays^8,24,28^. For example, Ismail and Shimada^24^ employed a robot hand illusion and found embodiment effects were abolished when the delay between performed and viewed robot hand movement was 290 ms and larger. Agency on the other hand was somewhat reduced with a 290 ms delay, but was only completely abolished with a 590 ms delay. This suggests that temporal comparisons between visual and non-visual signals influence both embodiment and agency. However, the time window for matching these signals is wider for agency than it is for embodiment.

In contrast to these findings, Caspar *et al*.^5^ have shown that *both* embodiment and agency are similarly affected by a more complex type of visual movement congruency cue. In their study participants performed simple index finger movements and viewed a robotic hand, which made either matching index finger movements (congruent effector condition) or similar movements of the little finger (incongruent effector condition). The authors found that effector movement incongruence significantly reduced embodiment *and* agency. Based on these results, Caspar *et al*.^5^ suggest that agency is affected by a body effector-specific matching mechanisms between visual and non-visual signals.

Interestingly, however, Ebert and Wegner^29^ have previously shown that movement congruency can affect agency even without body-specific visual movement feedback. These researchers used a paradigm in which participants were asked to either push or pull a joystick. After different delays, participants then viewed images on the screen that moved in a direction that was either congruent or incongruent with their joystick movement. Movement direction incongruence significantly reduced agency and the authors concluded that action-event consistency is an important agency signal.

Together, the previous research findings by Caspar *et al*.^5^ and Ebert & Wegner (2010) show that movement congruency influences agency in the contexts of both body-specific as well as body-non-specific visual movement feedback. This suggests that agency is affected by a general mechanism that involves comparisons between visual and non-visual movement signals. In other words, it seems that in these cases a general mechanism compares internal and/or proprioceptive information associated with the generation of a movement with visual feedback about that movement. This matching mechanism may however be modulated by body-specific cues. To test this, it is necessary to compare the effect of movement congruency across both body-specific and body-non-specific contexts. Thus, the aim of our study, was to investigate the effect of movement congruency on agency in the context of viewing a realistic body form as well as when viewing a non-body object. We employed a virtual hand setup^30^ to test the impact of two bodily self-cues on agency. First, we manipulated viewed object *form*: participants either viewed a virtual hand or a virtual sphere movement as they were moving their own hidden finger. Second, we manipulated viewed *movement congruency*: the virtual object moved either in the same direction or the opposite direction as the participant’s actual finger, as they made a button press movement.

To quantify embodiment we used standard RHI scale items^3^. To quantify agency, in line with the previous studies^5,29^, we employed an implicit agency measure known as the “intentional binding” effect. Implicit measures index agency indirectly by investigating how agency cues affect other perceptual tasks. The intentional binding effect is a compression of the perceived temporal interval between an action and its outcome^31^. We also employed an explicit measure of agency. Explicit agency measures involve a direct conceptual judgement, typically obtained using rating scales^4^. Elbert and Wegner (2010) measured explicit agency using a single scale item asking participants how much it felt that their action caused the external event. In contrast, Caspar *et al*.^5^ asked participants to rate experiences specific to the hand movements such as “I felt as if I was controlling the movements of the artificial hand”. However, these types of rating scales conceptualise agency as a unidimensional construct; that is, these measures imply that sense of agency is a single dimension that varies only in degree. Recent work however suggests that agency is better understood as being comprised of multiple distinct elements^4,32,33^. Here we investigated the influence of our chosen bodily self-cues using a standardised scale that assesses multiple components of the sense of agency^8,34^.

We predicted that both *form* and *movement congruency* would influence embodiment ratings. For agency, based on the combined findings of Caspar *et al*.^5^ and Ebert and Wegner (2010) we expected a main effect of *movement congruency* for implicit and explicit measures. Furthermore, if agency is modulated by body specific movement information, then we would expect a main effect of *form* or an interaction of *form* and *movement congruency*.

## Methods

### Participants

Thirty volunteers were recruited via the Macquarie University Cognitive Science Participant Register (18 female; age M = 20.97 years, SD = 3.64 years, range 18–32 years). All participants were right-handed, had normal or corrected-to-normal vision and received $15. This research was conducted in accordance with the ethical standards laid down in the 1964 Declaration of Helsinki and was approved by the Macquarie University Ethics Review Committee (Human Research). Written informed consent was obtained from all participants prior to the start of the experiment.

### Apparatus and Setup

**Figure 1:**
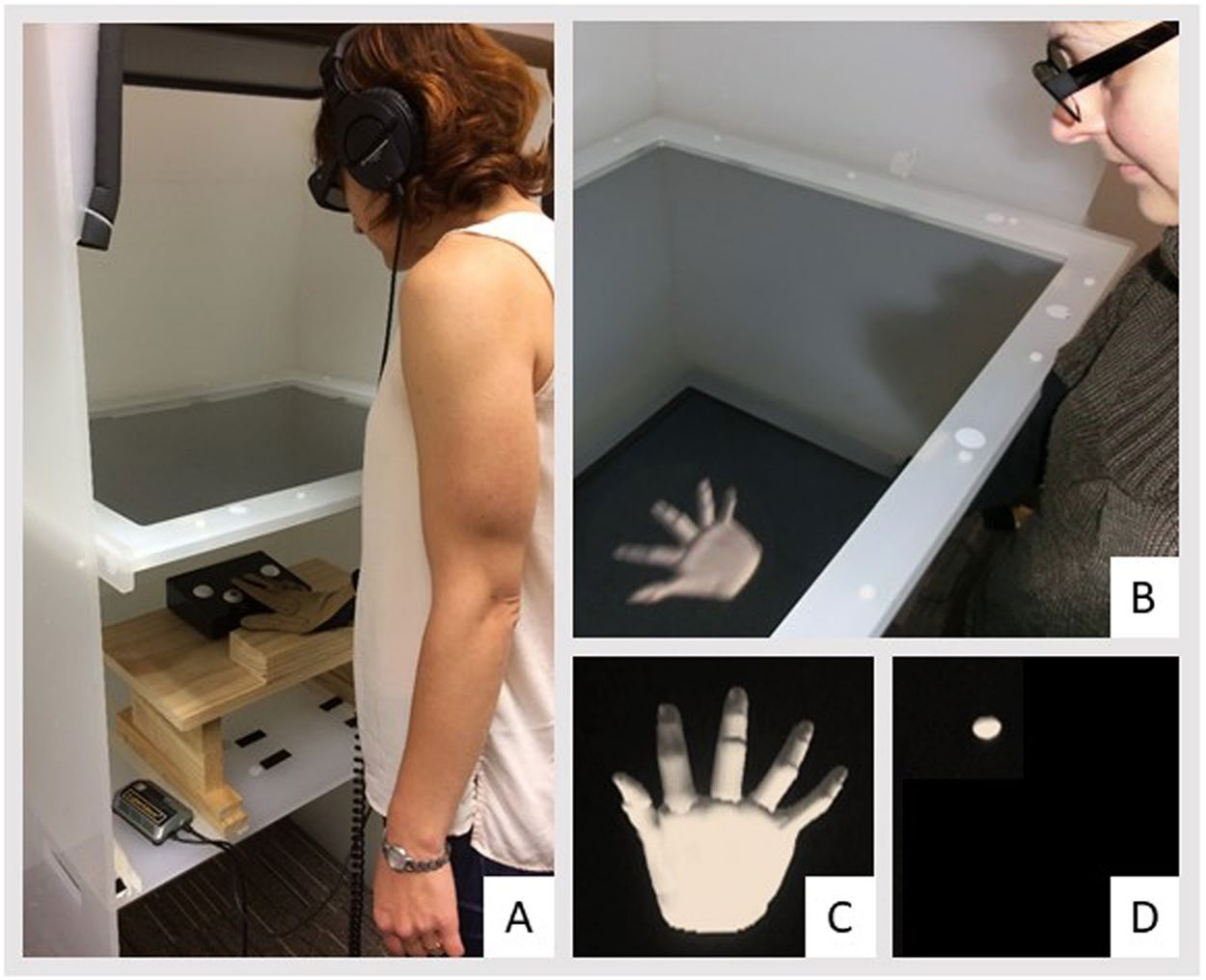
Virtual-hand and intentional binding task setup. (**A**) Participants wore a motion-capture glove and placed their hands under a mirror on a hand-rest. The participants’ index fingers were placed on top of a response button and participants wore headphones to listen to the tones of the intentional binding task. (**B**) Participants wore 3D glasses and viewed rendered hands (**C**) or spheres (**D**) at the same depth and location as the participants’ hidden hands.

To manipulate the visual feedback of performed finger movements, we used a virtual hand setup (Fig. 1). The setup consisted of a glove-based system (CyberGlove Systems) for capturing detailed hand movements, a magnetic motion capture system (Polhemus Fastrak) for capturing wrist movements, and a 3D screen (Hyundai TriDef, 60 Hz refresh rate) for visualizing movements. The 3D screen was mounted on top of a custom-built frame and participants viewed the screen’s images in a mirror that was mounted in the middle of the frame. The images appeared at approximately the same depth and location as the participants’ hidden hands which were placed on a hand-rest under the mirror. Participants wore polarized 3D glasses and the room was dimly-lit.

The software for collecting movement information and real-time rendering was custom-built (https://github.com/JasonFriedman/RepeatedMeasures) written in Matlab (MathWorks). The system delay for movement visualization was 80 ms^30^.

A separate computer running Presentation (Neurobehavioral Systems) was used to control stimulation and data collection for the intentional binding task. A response button was placed approximately 3 cm under the participants’ index fingers and participants wore headphones (Seinheisser HD280) for auditory stimulation.

### Design

**Figure 2:**
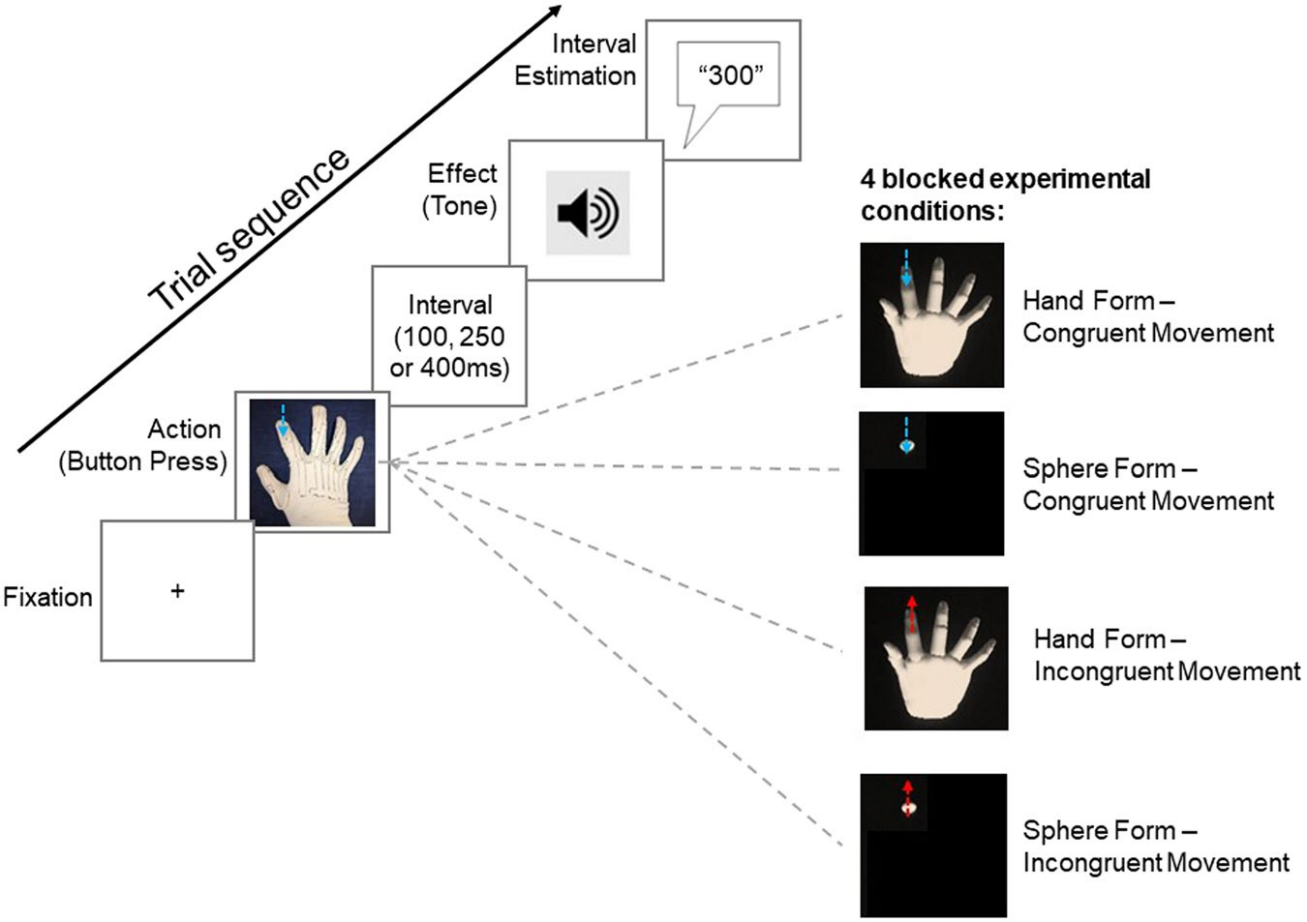
Trial structure and schema of experimental conditions. Each trial started with fixation. Participants then saw a rendered virtual object – either a hand or a sphere. Participants were asked to move the index finger down to press a button. The index finger of the virtual hand or sphere moved either congruently and downwards (blue arrow) or incongruently and upwards (red arrow). The button press was followed by a tone after an interval of either 100, 250 or 400 ms. Participants were then asked to estimate the interval between button press action and consequent tone.

We implemented a 2 × 2 within-participant-design and manipulated two bodily self-cues –*form* and *movement congruency* – during this task (see Fig. 2 for overview experimental conditions). For the visual form manipulation, either a virtual hand or a virtual sphere was visible. The virtual sphere corresponded to approximately the same size and location as the tip of the index finger. For the movement congruency manipulation, the displayed movement of the virtual hand or sphere was either congruent and in the same direction to the movement of the participant’s actual index finger (i.e., a downward finger movement to press the response button resulted in the displayed finger or sphere image moving downward at the same time) or the displayed movement was incongruent and in the opposite direction to the movement of the participant’s index finger (i.e., a downward finger movement to press the response button resulted in the displayed finger or sphere image moving upward at the same time).

### Measures

#### Embodiment ratings

We used two modified ratings scale items that have been used to investigate embodiment changes especially related to the embodiment subcomponent “ownership” in the rubber hand illusion paradigm^3^. These items were: Q1) “I felt as the 3d image was my hand” and Q2) “I felt as if the virtual hand/dot display was part of my body”. These ratings were used as a manipulation check to confirm that our experimental manipulations did indeed change the sense of embodiment. Participants completed embodiment ratings on a 7-point Likert scale ranging from 1 (‘strongly disagree’) to 7 (‘strongly agree’) after the agency ratings (i.e., four times in total).

#### Intentional binding task

We used an implicit agency measure known as the intentional binding paradigm. We implemented the interval estimation version of the intentional binding task because it is well-suited to measuring intentional binding effects in tasks that involve visual stimuli^5,35^. The structure of each trial in the interval estimation intentional binding task is shown in Fig. 2. Participants first saw a fixation cross in the centre of the screen for 1500 ms. The fixation cross then disappeared and participants saw a virtual display with a rendered hand or sphere (depending on the experimental condition, details of conditions below). This virtual representation responded to movements of the participant’s actual hand throughout each trial. Participants were instructed to move their index finger down and press a response button at a time of their choosing. The response button triggered an auditory tone (1000 Hz for 100 ms duration) after a delay. Participants were told that the interval would randomly vary between 1 and 500 ms. In reality there were only three possible delay intervals: 100 ms, 250 ms and 400 ms. After the tone, participants estimated the interval between their action (button press) and the start of the consequent tone. Participants were asked to report numbers in the range from 1 to 500 ms. Participants verbally reported their estimate and this was recorded by the experimenter. Participants were asked to rest their index finger above the response button at the end of each trial. There were four blocked experimental conditions and each delay interval was repeated 20 times resulting in 60 randomly-presented trials per condition.

#### Agency ratings

To measure explicit agency experience, we used the Sense of Agency Rating Scale (SOARS)^34^. SOARS has two factors: Involuntariness, which represents a subjective reduction in control over one’s own actions (e.g., “I felt that my experiences and actions were not caused by me”); and Effortlessness, which represents a subjective increase in the ease and automaticity with which actions occur (e.g., “My experiences and actions occurred effortlessly”). This scale was originally developed to measure general agency changes in the context of hypnosis but we used a modified version with general wording applicable to any context^8^. The SOARS requires participants to rate their level of agreement with a series of 10 statements using a 7-point Likert scale ranging from **1 (**‘strongly disagree’) to **7 (**‘strongly agree’). Participants completed a pen and paper copy of this scale after each experimental block (i.e., four times in total).

### Procedure

Participants put the data glove on their right hand, which was then placed onto a hand support under the mirror display. Participants were asked to respond in the intentional binding task by pressing the button with finger-flexion movements that were approximately 3 cm in depth. After each movement participants positioned their finger above the response button as they waited for the next trial to begin. Participants completed four blocks of the intentional binding task. We blocked the conditions because we were concerned that sudden changes in form or movement congruency could lead to surprise and affect the intentional binding measure. Blocking the trials also allowed us to probe changes in explicit ratings of embodiment and agency after each block. At the completion of each block participants filled out the SOARS scale and embodiment ratings, reporting their experiences in the preceding block. The order of conditions was counterbalanced across participants. The experiment took 60 minutes to complete.

### Statistical analysis

For explicit embodiment and agency rating analyses we conducted 2 *form* (hand versus dot) × 2 *movement congruency* (congruent versus incongruent) ANOVA on Embodiment items Q1 and Q2, as well as SOARS Involuntariness and SOARS Effortlessness ratings. We found non-normal rating response distributions for some rating scales (Shapiro-Wilk tests, p < 0.05). However, ANOVA are robust also for non-normally distributed data when the sample size is equal^36^.

To analyse the intentional binding data, we conducted a 2 (*form*: hand versus dot) × 2 (*movement congruency*: congruent versus incongruent) × 3 (*interval*: 100 ms versus 250 ms versus 400 ms) repeated measures ANOVA on participants’ interval estimates. Finally, we conducted correlation analyses to investigate the associations between intentional binding effects and explicit measures of embodiment and agency.

### Data availability

The datasets analysed during the current study are available from the corresponding author on reasonable request.

## Results

### Embodiment ratings

**Figure 3:**
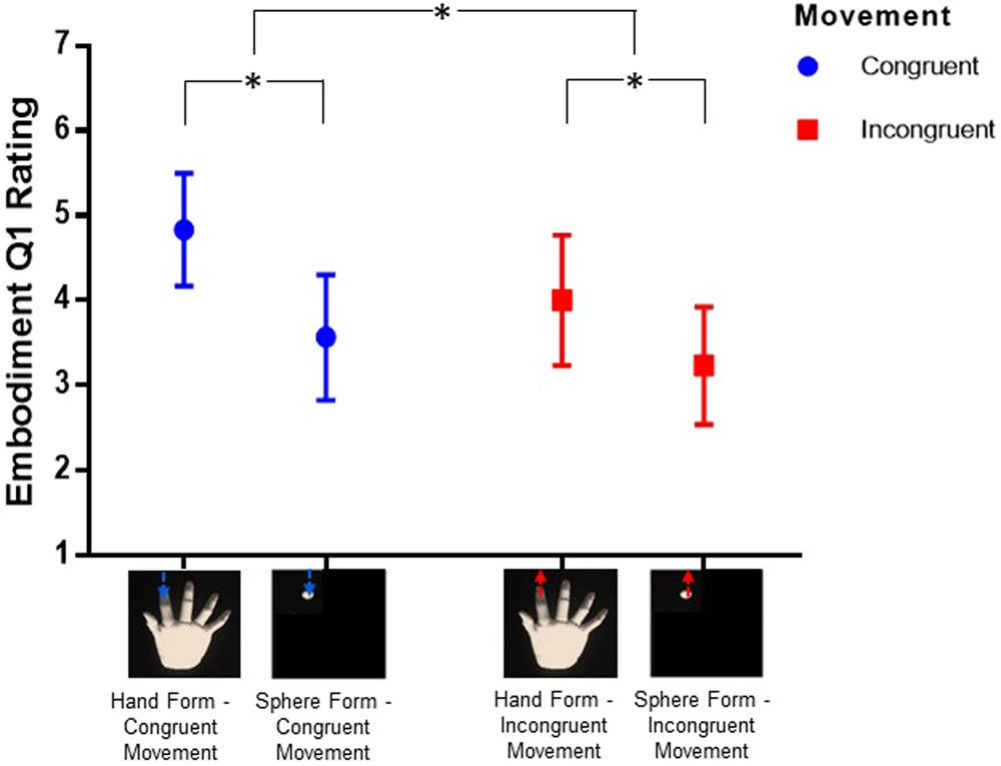
Embodiment ratings (Q1) for form and movement congruency conditions. We found a main effect of Form, F(1, 29) = 16.13, p < 0.001. Explicit embodiment was rated higher in the hand compared to the sphere conditions. There was also a main effect of movement congruency, F(1, 29) = 4.97, p = 0.034, with higher ratings for congruent (blue circles) compared to incongruent movements (red squares). Bars represent 95% confidence intervals.

We first analysed participants’ embodiment ratings to ensure that our manipulations did lead to changes in embodiment experiences. There were two embodiment rating scale items: Embodiment item Q1 – “I felt as if the 3d image was my hand” and Q2 – “I felt as if the virtual hand/dot display was part of my body”. As expected, for Q1 the ANOVA revealed a main effect of *form* (*F*[1, 29] = 16.13, *p < *0.001, *η*_*p*_^2^ = 0.357) with higher embodiment ratings for the hand conditions (*M* = 4.42, 95% CI [3.80, 5.04]) compared to the sphere conditions (*M* = 3.40, *95% CI* [2.81, 3.99], see Fig. 3). There was also a main effect of *movement congruency* (*F*[1, 29] = 4.97, *p* = 0.034, *η*_*p*_^2^ = 0.146) with higher ratings for congruent (*M* = 4.20, *95% CI* [3.61, 4.79]) compared to incongruent movements (*M* = 3.62, *95% CI* [2.99, 4.25]). There was no interaction of *form* and *movement congruency* for Q1 (*F*[1, 29] = 0.89, *p* = 0.352, partial *η*_*p*_^2^ = 0.030). Neither f*orm* or *movement congruency* had a significant impact on Q2 ratings (all *p* > 0.05).

### Implicit Sense of Agency: Intentional Binding

**Figure 4:**
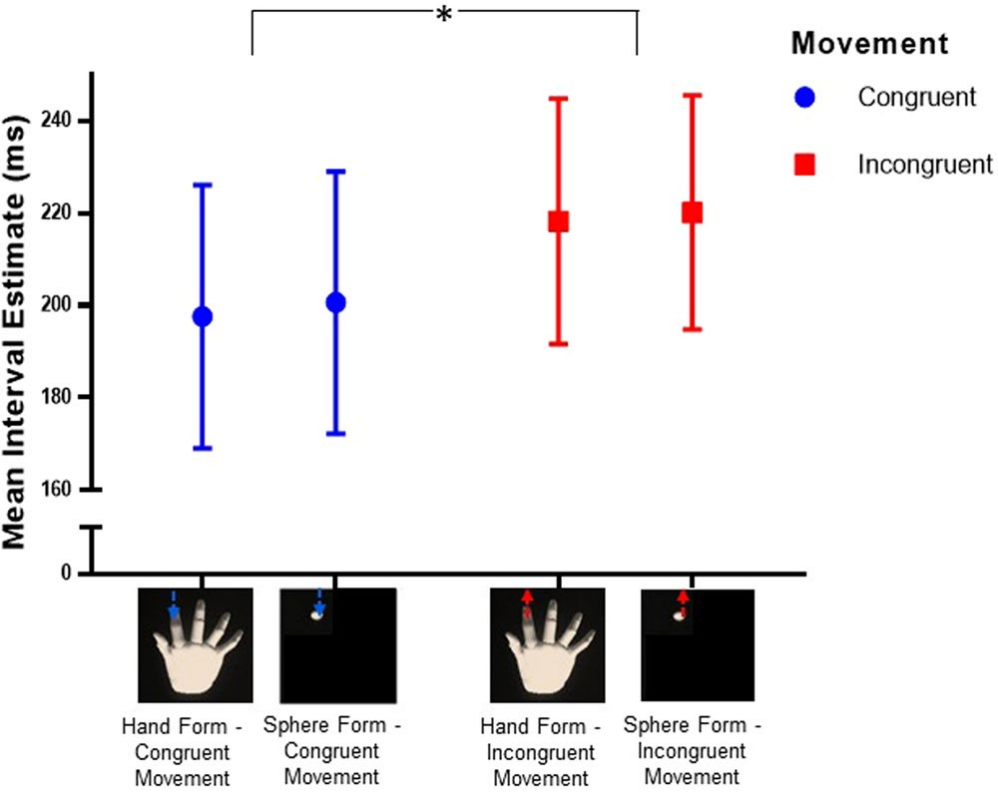
Interval estimates for form and movement congruency conditions. The interval estimates are averaged across all three presented interval durations (i.e., 100, 250, and 400 ms). We found a main effect of movement congruency, F(1, 29) = 6.16, p = 0.019. Intentional binding was reduced (larger time estimates) in the incongruent (red squares) compared to the congruent condition (lower time estimates, blue circles). Bars represent 95% confidence intervals.

Our main measure of interest was intentional binding scores, as an implicit sense of agency measure (Table 1; Fig. 4). An unsurprising main effect of *interval* (*F*[2, 58] = 66.12, *p* < 0.001; *η*_*p*_^2^ = 0.695) indicated that participants’ estimates increased with increased interval conditions. Importantly, we found a main effect of *movement congruency* (*F*[1, 29] = 6.16, *p* = 0.019, *η*_*p*_^2^ = 0.18) such that participants’ overall estimates were lower when movements were congruent (*M* = 199.13, *95% CI* [172.98, 225.28]) compared to when they were incongruent (*M* = 219.22, *95% CI* [195.96, 242.48]). *Form* did not significantly influence intentional binding estimates (*F*[1, 29] = 0.12, *p* = 0.730, *η*_*p*_^2^ = 0.004) and there was no significant interaction of *form* and *movement congruency* (*F*[1, 29] = 0.005, *p* = 0.947, *η*_*p*_^2^ < 0.001). There were also no significant interactions with *interval *(all *p* > 0.05).

**Table 1:** Average interval estimates for each experimental condition at each interval duration.

Movement	Form	100 ms		250 ms		400 ms	
		Mean (in ms)	95% CI	Mean (in ms)	95% CI	Mean (in ms)	95% CI
Congruent							
	Hand	145	115–175	190	162–218	258	222–294
	Sphere	141	114–167	197	165–229	264	227–302
Incongruent							
	Hand	164	134–193	216	187–244	275	244–306
	Sphere	159	131–186	219	195–244	283	248–317

CI, 95% Confidence Interval.

### Explicit Sense of Agency: Sense of Agency Rating Scale

The SOARS scale provided an explicit measure of general agency experience. We found a main effect of *form* for SOARS Effortlessness (*F*[1, 29] = 4.34, *p* = 0.046, *η*_*p*_^2^ = 0.130), with greater effortlessness in the sphere condition (*M* = 24.37, *95% CI* [22.92, 25.81]) than in the hand condition (*M* = 23.45, *95% CI* [22.09, 24.81]). There was no significant main effect of *movement congruency* (*F*[1, 29] = 1.72, *p* = 0.20, *η*_*p*_^2^ = 0.056) and no significant interaction of *form* and *movement congruency* (*F*[1, 29] = 0.05, *p* = 0.82, *η*_*p*_^*2*^ = 0.002). There were no significant effects for the SOARS Involuntariness scale (all *p* > 0.05).

### Correlations implicit and explicit measures

We next looked at associations between intentional binding and explicit measures of embodiment and agency. As *movement congruency* was the only factor that influenced binding scores we calculated the difference between congruent and incongruent movement conditions, collapsed across *form*, for each variable (binding scores, SOARS Involuntariness SOARS Effortlessness, Embodiment items Q1 and Q2). This allowed us to explore whether the effect of movement congruency correlated across these measures. There were no significant associations between the effects of congruency on intentional binding and explicit measures of embodiment and agency (binding scores – SOARS Involuntariness: *r* = −0.177, *p* = 0.348; binding scores – SOARS Effortlessness: *r* = 0.143, *p* = 0.451; binding scores – Embodiment item Q1: *r* = −0.007, *p* = 0.970; binding scores – Embodiment item Q:2 *r* = −0.178, *p* = 0.348). Furthermore, we also found no significant associations between the effects of congruency on explicit measures of agency and explicit measures of embodiment (SOARS Involuntariness – Embodiment item Q1: *r* = −0.094, *p* = 0.621; SOARS Involuntariness – Embodiment item Q2: *r* = −0.248, *p* = 0.187; SOARS Effortlessness – Embodiment item Q1: *r* = 0.097, *p* = 0.609; SOARS Effortlessness – Embodiment item Q2 *r* = 0.125, *p* = 0.509).

## Discussion

We investigated the effects of form and movement congruency on embodiment and agency using an intentional binding task. Binding was significantly affected by the congruency of visual and non-visual movement signals. When visual and non-visual movement information was congruent (i.e., a downward movement was viewed when the index finger moved downward to press the button) binding increased (i.e., interval estimates were lower) compared to when movement signals were incongruent (i.e., an upward movement was viewed when the index finger moved downward to press the button). This finding is in line with previous research that suggests that the matching of movement type is an important factor for implicit agency^5,29^.

However, body form plausibility did not modulate the effect of movement congruency on implicit agency. In other words, we found no evidence that the movement congruency effect is different in body-contexts compared to non-body contexts. This suggests that the movement type matching mechanism between visual and non-visual signals that modulates agency is not body-specific as has been previously suggested by Caspar *et al*.^5^.

We also found no significant main effect of *form* on binding. This suggests that implicit agency does not depend on the viewed virtual object being a visually plausible representation of one’s own hand. This is in line with the previous finding that anatomical plausibility with respect to viewed hand orientation (i.e., viewing a hand in an upright and anatomical plausible orientation versus a hand turned by 180° and anatomical implausible orientation) does not modulate implicit agency^9^. Thus overall, there is consistent evidence that the anatomical plausibility of visual information for one’s own body and thus viewing movement within a body-context does not modulate implicit agency.

In contrast, our study supports previous work showing that the anatomical plausibility of visual information for one’s own body influences embodiment and also explicit agency measures^6,8–10^. Although here, in contrast to our previous work^8^, we surprisingly found that viewing a body form decreased effortlessness (i.e., increased agency), compared to viewing a sphere. It may be possible that because the sphere was smaller than the hand, tracking movements was harder (took more effort) in this condition. Overall, although there is consistent evidence that anatomical plausibility does not modulate implicit agency, it appears that anatomical plausibility may modulate explicit agency. It may be possible that explicit agency judgements are more likely influenced by participants’ intuitions and expectations about the link between their own bodies and their perceived action control.

Intriguingly we did not find an effect of *movement congruency* on our explicit agency measure. This finding is inconsistent with previous work suggesting that *movement congruency* also modulates explicit agency^5,29^. It’s possible that this is because our agency rating scale was too broadly focused on the general phenomenology of causality. The SOARS scale has been used to discriminate agency experiences related to different types of hypnotic suggestion^32^ and different types of clinical symptoms in schizophrenia^37^. It may be that although this measure is able to tap high level cognitive attributions about agency in different contexts, it is less sensitive to the low level sensorimotor movement cues used in this study.

As expected, we did find a significant effect of *movement congruency* and *form* for the embodiment item Q1 (“I felt as if the 3d image was my hand”). This item is thought to be a core indicator of embodiment in the rubber hand illusion paradigm^3,27,38^. Furthermore, we found no interaction between the two bodily-self cues *movement congruency* and *form*. This is in line with previous work manipulating *synchrony* and *form* in both movement-based^10^ and touch-based^8^ virtual versions of the rubber hand illusion paradigm and suggests that each of these bodily-self cues does not constraint the influence of the other. Surprisingly, we did not find significant effects for item Q2 (“I felt as if the virtual hand/dot display was part of my body”). This item has been less commonly used in the rubber hand literature, and the null effect here might be due to the fact that in our study the virtual object was presented without implied connection to one’s own body (i.e., participants viewed a hand disconnected from any arm). The perceived connection of external objects has been shown to modulate embodiment illusions^22,39^ and might be more relevant for some embodiment items than others. One limitation in our work was that we did not employ an implicit embodiment measure such as the perceived hand position^5,6,27,40^. However, movement congruency and form have both consistently been found to modulate both explicit and implicit embodiment in previous studies^5,41^.

Considering these results together with the previous literature, what can we infer about the links between agency and embodiment mechanisms? First, consistent with previous research, our measures of implicit and explicit self-perception differed at least to some extent^4,29,42–44^. Explicit and implicit measures for self-representation are typically affected by the same cues, however the relative influence of specific cues can also differ across measures as well as task contexts. This suggests (at least partly) independent mechanisms for implicit and explicit aspects of self-representation^42–44^.

Second, both implicit and explicit measures of agency and embodiment were modulated by the comparison of visual and non-visual movement signals. In this study, and in previous work, movement congruency influenced all measures of self-perception^5,29^. This supports a view that both agency and embodiment mechanisms operate by making comparisons across different signals. These comparisons might involve internal and/or proprioceptive information associated with the generation of a movement with visual feedback about that movement. Furthermore, this matching mechanism does not necessarily have to involve a spatio-temporal pattern of sensorimotor signals, but instead could involve the matching of intended action goals and perceived events^4^. In other words, instead of comparing the low-level visual and non-visual movement direction cues, the matching mechanism might compare higher-level representations such as ‘upward’ versus ‘downward’.

Third, embodiment mechanisms may be predominately influenced by spatio-temporal matching of low-level body-related signals. Whereas agency could depend to a greater extent on higher-level mechanisms that compare more general representations of intended action goals and perceived events^45,46^. Support for this idea comes from our and previous results showing that, in contrast to agency, embodiment is more strongly affected by visual form and anatomical plausibility cues^6^ and subject to stricter temporal constraints^24^. Furthermore, it is notable that we did not find significant correlations of movement congruency effects across different measures. Overall this suggests that the comparison mechanisms differ (at least to some extent) for implicit and explicit agency and embodiment, but that on a general level all mechanisms involve comparisons across different (e.g., visually and non-visually derived) signals.

In conclusion, we found that movement congruency enhances implicit agency and that this effect does not depend on the presence of body-specific signals such as viewing a body form in an anatomical plausible orientation. In contrast, both movement congruency and the anatomical plausibility of viewed body information are important factors for embodiment mechanisms. On a more general level, our findings suggest that both implicit and explicit agency and embodiment mechanisms depend on comparison mechanisms that match different types of signals. These comparisons however seem to be influenced by distinct factors and spatio-temporal boundaries which suggests that they involve different types of representations.
